# How to improve the quality of care for people on home mechanical ventilation from the perspective of healthcare professionals: a qualitative study

**DOI:** 10.1186/s12913-021-06743-3

**Published:** 2021-08-05

**Authors:** Hanna Klingshirn, Laura Gerken, Katharina Hofmann, Peter Ulrich Heuschmann, Kirsten Haas, Martha Schutzmeier, Lilly Brandstetter, Jutta Ahnert, Thomas Wurmb, Maximilian Kippnich, Bernd Reuschenbach

**Affiliations:** 1 Catholic University of Applied Sciences Munich, Preysingstraße 95, 81667 Munich, Germany; 2 Institute for Clinical Epidemiology and Biometry, Julius-Maximilian University Würzburg, Josef-Schneider-Straße 2, 97080 Würzburg, Germany; 3 Clinical Trial Center Würzburg, University Hospital Würzburg, Josef-Schneider-Straße 2, 97080 Würzburg, Germany; 4 Comprehensive Heart Failure Center Würzburg, University and University Hospital Würzburg, Am Schwarzenberg 15, 97078 Würzburg, Germany; 5 Department of Anaesthesiology, Intensive Care, Emergency and Pain Medicine, University Hospital Würzburg, Oberdürrbacher Straße 6, 97080 Würzburg, Germany

**Keywords:** Home mechanical ventilation, Quality of care, Person-centred care, Healthcare professionals, Qualitative study

## Abstract

**Background:**

The rapid increase in the use of home mechanical ventilation (HMV) for people with chronic respiratory failure poses extreme challenges for the healthcare system. People on HMV have complex care needs and require support from an interprofessional team. In Germany, HMV is criticised for inadequate quality standards, particularly in outpatient intensive care practice. The objective of this study was to describe the quality of care for people on outpatient HMV in Germany, Bavaria and provide recommendations for improvement from the perspective of healthcare professionals (HCPs).

**Methods:**

Semi-structured qualitative telephone interviews with HCPs (i.e., nurses, equipment providers, therapists, and physicians) were analysed using the framework method. The quality framework of Health Improvement Scotland (HIS), which aims to improve the quality of person-centred care, was used to build a deductive analysis matrix. The framework includes the three key areas: (1) *Outcomes and impact*, (2) *Service delivery*, and (3) *Vision and leadership*. The domains (meta-codes) and quality indicators (sub-codes) of the quality framework were used for deductive coding.

**Results:**

Overall, 87 HCPs (51 female, mean age of 44.3 years, mean professional experience in HMV of 9.4 years) were interviewed (mean duration of 31 min). There was a complex interaction between the existing health care system (*Outcomes and impact*, 955 meaning units), the delivery of outpatient intensive care (*Service delivery*, 939 meaning units), and improvement-focused leadership (*Vision and leadership*, 70 meaning units) that influenced the quality of care for people on HMV. The main barriers were an acceleration in transition management, a neglect of weaning potential, a shortage of qualified professionals and missing quality criteria. The central recommendations for promoting person-centred care were training and supervision of staff and an inspiring leadership. An integrated care structure supporting medical home visits and outpatient rehabilitation should be developed.

**Conclusion:**

This study describes a heterogeneous and partly deficient care situation for people on HMV, but demonstrates that high quality care is possible if person-centred care is successfully implemented in all areas of service provision. The recommendations of this study could inform the development of a person-centred integrated care structure for people on HMV.

**Supplementary Information:**

The online version contains supplementary material available at 10.1186/s12913-021-06743-3.

## Background

In recent decades, the use of home mechanical ventilation (HMV) has increased worldwide, and HMV has evolved into a well-established treatment option for people with chronic respiratory failure [1]. The population of people on HMV support is extremely heterogeneous, and individual care needs are complex and vary greatly. Chronic respiratory failure can occur in people with chronic obstructive pulmonary disease (COPD), obesity hypoventilation syndrome, thoracic-restrictive lung disease, or neuromuscular disorder. Mechanical ventilation treatment depends on the underlying disease and can be performed invasively (e.g., via tracheal tubes) or non-invasively (e.g., via face masks) [1, 2].

Currently, non-invasive ventilation is indicated in considerably more people than invasive ventilation via tracheotomy [1]. In the 2005 Eurovent study [3], the estimated prevalence of HMV per 100,000 population was 6.6 for Europe. Recent studies from Poland [4], Canada [5], and Germany [6] show that the number of people on HMV has increased rapidly over time. In Germany, the number of long-term ventilated patients who had to be re-hospitalised for ventilation control or emergency more than tripled between 2006 and 2016 (from 24,845 to 86,117 patients). In addition to chronic respiratory failure, these patients were characterised by a variety of internal and neurological comorbidities and could therefore be considered a severely ill population [6].

Since caring for people on HMV requires high professional and technical expertise, the rapid increase in the use of HMV is changing the nursing landscape and posing extreme challenges to the healthcare system. Practices and policies regarding HMV are quite different across countries [7]. In Germany, HMV is provided in either an inpatient intensive care facility or outpatient supported by an intensive home care service or a personal assistant. Outpatient intensive care can be provided either in the persons’ private home or in a specialised shared living community. The decision about the care setting is made by the affected person (or the persons’ legal guardian) and is influenced by the severity of the disease, the complexity of care needs, and structural requirements (e.g., accessibility, the regional availability of support services) [8]. An interprofessional team of nurses, physicians, equipment providers and therapists is recommended to meet the complex care needs of people on HMV [8]. An effective and structured collaboration between the patients, their relatives and the healthcare professionals (HCP), requires a care network that ensures outpatient care and integrates expertise from specialised ventilation centres using a cross-sectoral approach [8–10].

There is international consensus that the establishment of a high-quality HMV has the potential to improve health-related quality of life and to reduce hospitalisations but is associated with a high burden for family caregivers (e.g., emotional, financial) [7]. Despite the positive impacts of HMV treatment, its increasing prevalence has a strong impact on routine care and healthcare system sustainability, as HMV is extremely resource- and cost-intensive [11, 12]. In Germany, it has been criticised that financial disincentives, inadequate quality standards, and a lack of control mechanisms lead to mismanagement in the care of people on HMV. The main concern is that there are no incentives to promote rehabilitation, especially weaning from ventilation [13, 14]. Internationally, there is little evidence on how HMV service provision should be delivered to improve person-centred outcomes [7]. Recent German studies indicate the importance of understanding the complexity of healthcare needs and identifying necessary actions to improve the quality of care for people with HMV across multiple sectors and professions [10, 15]. Based on this need, we started the *Optimising the Care of Ventilated Patients in Outpatient Intensive Care* (OVER-BEAS) project, which aims to examine the quality of care for people with invasive and non-invasive mechanical ventilation in outpatient intensive care in Germany, Bavaria. Since quality of care in HMV includes the initiation, adaptation and control of HMV, the OVER-BEAS project also examines the quality of collaboration between the inpatient and outpatient sector [16].

### Objective

As part of the OVER-BEAS project, the objective of the present study was to describe the quality of care for people on HMV (invasive and non-invasive) in the outpatient setting (peoples’ private home or shared living community) from the perspective of HCPs. Moreover, we aimed to provide recommendations to improve the quality of care for people on HMV in Germany, Bavaria. The results of this study will be integrated in the development of interventions to support a successful person-centred care practice for people on HMV.

## Methods

### Study design

The framework method [17] and a deductive approach [18] were used for qualitative analysis. This method belongs to the family known as thematic analysis or qualitative content analysis. In the analysis process, the framework creates a structure for the collected data that is supportive in summarising the content of the interviews and thus in answering the research question. The framework method is a systematic approach often used for semi-structured interviews in health research [17]. As a theoretical structure for building an analysis matrix, we used the nine domains and 25 quality indicators of the Health Improvement Scotland (HIS) quality framework [19]. The outline structure of the quality framework is presented in Fig. ﻿1. We used the Standards for Reporting Qualitative Research (SRQR) to structure this paper and to improve the transparency of our research [20].

**Fig. 1:**
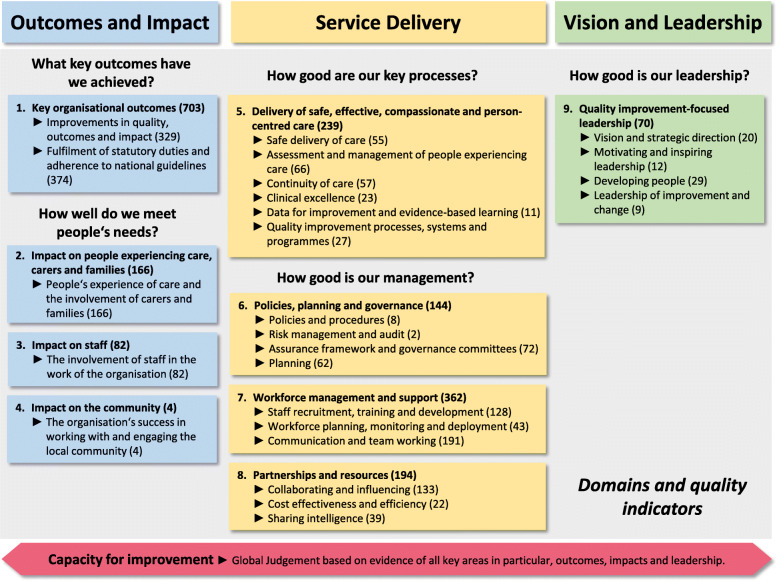
Outline structure of the HIS quality framework adapted from Health Improvement Scotland (2018) [19]. The numbers in brackets represent the meaning units per domain or per quality indicator

### The HIS quality framework

The HIS quality framework [19] aims to support the evaluation of existing governance structures and their influence on systems and procedures to ensure the delivery of safe, effective, compassionate and person-centred care. The HIS framework has been developed as a tool for self-evaluation and external quality assurance; it includes nine domains and 25 quality indicators to improve the quality of care. The domains can be assigned to three key areas:

I. *Outcomes and impact* describe the impact that statutory requirements of the health care system have on care, carers and people experiencing care over time.
II. *Service delivery* describes the quality of key processes and management structures to deliver safe, effective, compassionate and person-centred care.
III. *Vision and leadership* describe the quality of leadership and its impact on improvement.

The definitions and themes of each quality indicator are presented in our codebook in Additional file 1.

### Participants and setting

Participants were recruited from a convenience sample (i.e., existing expert network involved in the development of quality indicators as part of the OVER-BEAS project) and from intensive care services involved in the OVER-BEAS project. Snowball sampling (i.e., existing study participants recommend further potential study participants from their network) was used to identify further participants. We provided project flyers with study information to invite potential participants to complete a 15- to 30-min semi-structured interview. Study participants had to be at least 18 years old, be working in Bavaria (Germany), and be working as an HCP with at least 2 years of experience in supporting people on outpatient HMV (i.e., at home or in a shared living community). This definition includes HCPs working in an inpatient setting, if they were involved in the initiation, adaptation and control of HMV. HCPs were defined as nursing managers (i.e., managing directors of home intensive care services), head nurses, registered nurses, nursing experts (i.e., nursing instructors, consultants, or auditors), equipment providers, physiotherapists, occupational therapists, speech and language therapists, and physicians. This interprofessional approach was chosen to reflect the different views of all stakeholders and to examine collaborative working across sectors.

### Data collection

Data were collected from June 2019 to May 2020. Interviews were conducted via telephone by two trained interviewers (KH and BH). All interviewers had a therapeutic or nursing background and were familiar with the field of HMV and its practical conditions. This was important to create an interview situation in which interviewers and HCPs could exchange knowledge and information as equal partners. The participants’ characteristics (i.e., gender, age, occupational group, and years of professional experience) were collected in the first part of the interview, and field notes (i.e., on the interview duration and special incidents) were taken following the interview. The participants were provided with the key questions from the interview guide in advance so that they could be prepared for the interview.

### Ethical considerations

Informed written consent was obtained from all participants before the study was conducted. To ensure that none of the HCPs felt coerced to participate in the study (some HCPs were recruited via their employers e.g., intensive care services), special reference was made to the voluntary nature and anonymity of participation. Additionally, all participants provided verbal consent prior to the audio-recording of the interviews. The study was approved by the local ethics committee of the Catholic University of Applied Sciences Munich.

### Interview structure

The interview structure is presented in Table 1. The interview guide was pretested with one HCP before data collection started but needed only minor adjustments (e.g., addition of follow-up questions regarding the specific care setting, treatment goals and financing). Before the interviews, the participants were encouraged to present their personal views of the care situation for people on HMV support. After an opening question (i.e., introduction of the participant), key questions were asked with a focus on the following four areas: (1) daily care practice, (2) quality of care, (3) collaboration, and (4) capacity for improvement. These four areas were chosen to present a broad view of the actual care situations of people on HMV. Each individual area in turn represented different domains of the quality framework [19], which enabled us to adopt a holistic perspective. During the interview, follow-up questions or probes (i.e., further explanations or examples) were offered to draw out additional information when necessary. To close the interview, the participants were asked if they had anything else they would like to share.

**Table 1:** Interview structure

Focus	Key questions	Follow-up questions
Daily care practice	Please describe your daily care practice with people on HMV as concretely as possible.	What other activities do you carry out?
		What goals define your professional behaviour?
		Please describe a concrete example of the care process.
Quality of care	How do you perceive the quality of care for people on HMV? What is beneficial for a successful care practice?	What skills and qualifications are necessary to care for people on HMV?
		How do you implement your quality expectations in daily practice? What are barriers and facilitators?
		What is the role of financing?
Collaboration	What are factors of a successful interprofessional (or intersectoral) collaboration?	How is interprofessional (or intersectoral) collaboration organised? Which communication channels do you use?
		What works best? Where are the biggest problems?
Capacity for improvement	Where do you see a substantial need for improvement in the care for people on HMV?	Please summarise.

*Abbreviations: HMV* Home mechanical ventilation

### Data processing and analysis

All interviews were audio-recorded and transcribed verbatim. Personal data were pseudonymised to protect data. To enhance rigor [21], the participants were offered an opportunity to review the transcript. The data were analysed by two researchers (HK, MPH and LG, MSc), both of whom had a therapeutic or nursing background and were experienced in qualitative data analysis (i.e., both already conducted qualitative content analyses in previous research projects). The domains (meta-codes) and quality indicators (sub-codes) of the quality framework [19] were used to build an analysis matrix for deductive coding.

Two principles of saturation were used: code saturation or “the range of thematic issues” and meaning saturation or “the understanding of issues” [22]. Code saturation was used to determine the number of interviews that needed to be coded in parallel by the two independent researchers (HK and LG) until the HIS quality framework was exploited in the context of HMV.

We considered code saturation to be reached when every sub-code had been assigned two times and at least two interviews from each occupational group had been analysed. During parallel coding, all codes were compared and – in case of divergence – discussed until consensus was reached. Code saturation was reached after 43 interviews were parallel coded. Subsequently, the coding process was shifted to single coding (HK = 27, LG = 17), during which the independent researchers worked with memos to discuss uncertainties in the coding. The sample size was limited based on the principle of meaning saturation: As long as new themes emerged, additional HCPs were included in the study. Meaning saturation was reached after 87 interviews.

Data analysis was supported by MAXQDA software, version 20. A code map was created to visualise intersections of codes in a segment between the domains (meta-codes).

## Results

Overall, 87 HCPs described the quality of care for people on HMV. The mean interview duration was 31 min (range 12–88 min). Three interviews were repeated due to problems with the recording. Sixty-one participants reviewed their transcripts, and two of them made minor (i.e., non-content-related) corrections.

### Characteristics of the participants

Of the 87 participating HCPs, 51 (58.6%) were female. The mean age was 44.3 years (SD 11.2), and the mean professional experience supporting people on HMV was 9.4 years (SD 6.0). All HCPs were experienced in invasive and/or non-invasive ventilation and in outpatient intensive care (i.e., peoples’ private home and/or shared living community) and/or in the intersection between outpatient and inpatient care (i.e., initiation, adaptation and control of HMV). Including nursing managers (*n* = 11), head nurses (*n* = 19), registered nurses (*n* = 20), nursing experts (*n* = 8), equipment providers (*n* = 7), physiotherapists (*n* = 6), occupational therapists (*n* = 3), speech and language therapists (*n* = 9), and physicians (*n* = 4), the HCPs presented a broad interprofessional perspective on the care of people on HMV. The characteristics of HCPs are presented in Table 2.

**Table 2:** Healthcare professionals’ characteristics

N	87
Sex (female), n (%)	51 (58.6)
Age in years, M (SD)	44.3 (11.2)
Occupational group, n (%)	
Nursing manager	11 (12.6)
Head nurse	19 (21.8)
Registered nurse	20 (23.0)
Nursing expert ^a^	8 (9.2)
Equipment provider	7 (8.0)
Physiotherapist	6 (6.9)
Occupational therapist	3 (3.4)
Speech and language therapist	9 (10.3)
Physician	4 (4.6)
Professional experience MV in years, M (SD)	13.6 (9.2)
Professional experience HMV in years, M (SD)	9.4 (6.0)
Advanced education as respiratory therapist, n (%)	9 (10.3)

*Abbreviations: M* Mean, *SD* Standard deviation, *MV* Mechanical ventilation, *HMV* Home mechanical ventilation

^a^ Nursing experts include nursing instructors, consultants, and auditors

### Analysis of the interviews

In total, 1964 meaning units were coded within the quality framework. Among these meaning units, the three key areas were represented, with 955 meaning units for *Outcome and impact*, 939 meaning units for *Service delivery*, and 70 meaning units for *Vision and leadership*. The number of meaning units for each quality indicator ranged from two to 374 (see Fig. 1).

### The HIS quality framework

This section describes the key elements and themes that emerged from the analysis of the HCPs’ perceptions of the quality of care for people on HMV in the outpatient intensive care setting.

### I. Outcomes and impacts

#### *1. Key organisational outcomes*

Overall, the HCPs reported that quality of care in HMV is highly heterogeneous depending on the individual provider. Increasing demands and accelerated processes have led to a decrease in the quality of care over the last decade:

*[My impression in the last years is that] well-fed and clean is somehow sufficient. [...] It's about providing care that is not yet dangerous, [...] but it's no longer about providing rehabilitation and integration for these people. (Nursing expert, ID40, §40)*

The decrease in the quality of care already arises in the hospital. Due to the acceleration of complex processes, for example, in transition management, there is no time to promote rehabilitation and weaning from ventilation. In addition, the shortage of qualified, experienced and motivated staff (i.e., physicians, nurses and therapists) was reported as a serious problem across all healthcare sectors:

*So, let's start with the biggest problem: the changes in the hospital landscape. So, the staff shortage in the clinics, the short-term transfer of patients [...]. [The] patient has to leave as quickly as possible. [...] It's much faster, less detailed. (Equipment provider, ID22, §20-22)*

To counteract these problems, the HCPs recommended that the available expertise should be used in a resource-efficient manner and in line with peoples’ needs. Achieving such resource efficiency involves an urgent need for medical home visits to avoid hazardous transport and hospitalisation:

*The biggest problem [is] physician care [...]. The patient can't come to the physician's practice, that would be much too complex, this transport. And that means you need a home visit. And you need someone who is competent and knows about ventilation, swallowing disorders and all that. (Physician, ID57, §25)*

The HCPs describe a systemic problem as the reason for the existing quality deficits. Existing legal requirements for the care of people on HMV promote a system of mismanagement in the healthcare system (e.g., accelerated and premature discharges from hospital, refusal of claims, long waiting times, and a high level of bureaucracy). A physician illustrated the impact of misaligned incentives on the facilitation of peoples’ weaning-potentials as follows:

*The problem is that the care services have no interest in weaning because then the customer is gone. [...] The moment the patient is recovered, they [care services] don't earn any money, and that is a very bad structure. (Physician, ID58, §33-35)*

Finally, the HCPs criticised that the German national HMV guideline is not mandatory, and some recommendations are not feasible to implement due to structural deficits (e.g., insufficient number of weaning centres, insufficient structures for interprofessional and intersectoral collaboration). To improve key organisational outcomes the HCPs recommended the implementation of a new integrated care structure that enables networking and interprofessional collaboration.

#### *2. Impact on peoples experiencing care, carers and families*

The HCPs emphasised that person-centred HMV should be guided by the idea of assistance. This means that care should support autonomy, focus on individuals’ needs and preferences, and enable ventilated patients and their families to consider treatment options and make informed decisions:

*Outpatient intensive care has a lot to do with assistance, simply understanding that someone who is fully ventilated can go to a rock concert or a restaurant or something similar, in other words, to enable participation. That is part of this service. (Nursing manager, ID34, §47)*

Furthermore, the HCPs highlighted shared decision making between professionals and people receiving care as a central point within the transition process and in the choice of an appropriate long-term care setting. Therefore, the assessment of rehabilitation potential is just as important as ethical considerations – especially if the ventilated patient is unable to provide consent. To support the decision-making process, the HCPs recommended interprofessional case conferences involving all stakeholders:

*The transition to the outpatient area [...] has to be managed in a completely different way in the clinic. There is a need for a medical-ethical case conference; there is a need for a serious effort to ensure that the patient experiences rehabilitation first [...]. With real information for the relatives: What is [...] possible? How ill is [...] the patient? (Nursing expert, ID41, §83)*

#### *3. Impact on staff*

The HCPs highlighted the major difference between the two care settings in outpatient HMV: at home (i.e., where the nurse works alone on a 12-h shift) or in a shared living community (i.e., where the nurse works as part of a team). The HCPs described caring at someone’s home as often being experienced as burdensome, especially due to difficulties in role definition and conflicts with family members. A quote from a nursing expert illustrates this problem:

*Most treatments fail because of the psycho-social context. [...] The real issue is definitely dealing with family and relatives, the many conflicts between nurses and relatives. [...] A big shortcoming [...] is that there are [...] no structured offers like supervision. (Nursing expert, ID44, §21)*

In contrast, the HCPs reported that a higher nursing staff-patient ratio in outpatient intensive care is related to more time and less stress. Therefore, the HCPs assumed that this time resource could be used to deliver high-quality person-centred care.

#### *4. Impact on the community*

In Germany, HMV is hardly engaged with the local community. Therefore, the HCPs emphasised the integration of the community sector (e.g., networking, integrated care, independent counselling, and guidance of relatives) as a new resource:

*The relatives should have the possibility to have a service point in the community or at a local level, where they can get their information. (Occupational therapist, ID46, §87)*

### II. Service delivery

#### *5. Delivery of safe, effective, compassionate and person-centred care*

Due to its importance as a life and death issue, the HCPs defined safety as a key objective for the delivery of HMV. Consequently, intensive care services require a strong culture of safety, including standardised safety policies and procedures. Nevertheless, the HCPs reported that qualification deficits are the most important risk factors for the safety of people on HMV:

*[During 24-hour emergency service], you experience [...] a lot. Well, [...] 70 to 80 % are operating errors, and the rest are technical problems. [...] Equipment is often [incorrectly] installed. [...] The handling is [...] sometimes catastrophic. (Equipment provider, ID26, §32)*

Moreover, the HCPs argued that delivering person-centred care should be based on empowering ventilated patients to be completely involved in all decisions concerning their care and support:

*No two days are the same, that's for sure, because it's individual care. I also attach a lot of importance to that. The client determines his daily routine. [...] Whether he wants to be washed, whether he wants to get up, whether he would rather stay in bed, whether he wants to sleep, whether he wants to watch TV in bed, whatever his wish is. (Registered nurse, ID60, §16)*

Regarding barriers to a seamless journey through different settings, the HCPs reported (1) accelerated processes, (2) stressful hospital stays (e.g., as the assistance of the trusted nurse ends at the hospital door) and (3) complex and hazardous transports. The HCPs emphasised that successful collaboration between different organisations is possible if the involved stakeholders work together and share information.

Overall, the HCPs described HMV as a well-established treatment option with recognised standards and agreed-upon best practices. Unfortunately, these standards are not mandatory. As a main barrier, the HCPs criticised that statutory quality control systems are lacking and that the responsibility to implement quality assurance systems belongs solely to intensive care services.

#### *6. Policies, planning and governance*

The HCPs emphasised the importance of an operating infrastructure, enabling staff to integrate national expert standards and routine mechanisms in daily care practice. Furthermore, the HCPs described proactive risk management as an essential element of safety.

As a key problem, the HCPs emphasised that existing structural deficits in physician care have a strong impact on the delivery of intensive home care. Due to the shortage of physicians specialising in HMV, roles and responsibilities between nurses and physicians are becoming blurred. Although physicians are actually responsible for the coordination of care, nurses often take over this role. The HCPs indicated collaboration with high-qualified nursing experts (e.g., advanced practice nurses, respiratory therapists) as a key resource, which could fill the gaps in medical care through the delegation of medical services:

*I take over the work from the medical specialist, the respiratory physician and the intensive care physician [...], but always with their supervision. The respiratory therapist [...] needs a physician who delegates this and also needs supervision. (Nursing expert, ID41, §25)*

Moreover, the HCPs emphasised that planning and coordination of service delivery is a major challenge in HMV, especially when several processes run in parallel or when needs are only reimbursed at a flat rate. A quote from a head nurse illustrates the problems arising in this area:

*If you now need two tube extensions a day, the provider tells you to clean the tube extension. [...] I can't wash a disposable product with water that contains, for example, secretions and bacteria [...]. Then, the [patient] has pneumonia one day later. (Head nurse, ID4, §42-44)*

#### *7. Workforce management and support*

A major problem is the critical staffing situation in the healthcare sector. In this regard, the HCPs listed the recruitment, induction and training of staff as key indicators for the delivery of high-quality care. Optimally, these processes require a long induction period, the provision of advanced education, internal training and supervision:

*We [plan] a very strong and very long induction phase [...] so that everyone involved in care feels safe, i.e., the patient, relatives and, of course, the employee himself. [...] Then, we strongly focus on training and advanced education, which means that we train employees in outpatient respiratory care externally but also through regular quality trainings internally. (Head nurse, ID5, §19)*

In addition, the HCPs described that a high level of clinical expertise (e.g., experience and knowledge in critical care and respiratory care) is needed in caring for people on HMV, although the core competencies include monitoring, empathy, and social care. In this regard, the HCPs emphasised the importance of family-centred care, including professionally handling proximity and distance, involving the family, and being a guest in a person’s home:

*In the outpatient intensive care context, [...] the patient [is] not ill alone; [...] the relatives and families [must] be involved in the care [...]. And then it's a lot about the themes of proximity and distance; mindful, appreciative interaction with relatives and families; [...] feeling like a guest in the [patient's] home. (Nursing expert, ID44, §17)*

The HCPs described that in addition to nursing competence, the flexibility of the nursing team (e.g., skills mix, appropriate nurse-to-patient-ratio) is important in the care of people on HMV. Moreover, they described HMV as highly interprofessional. Therefore, the HCPs emphasised the need for effective communication among all internal and external stakeholders to ensure the achievement of common treatment goals:

*The great thing is [...] the interprofessional team; everyone contributes something new and says, “Watch out, we can take a look at that.” [...] I as a physiotherapist [...], the speech and language therapist [...], as well as the nursing staff [...] who are much closer to the patient, [...] you can involve [...] them as co-therapists. (Physiotherapist, ID52, §19)*

As a key problem, the HCPs emphasised that existing structures hamper communication with external stakeholders. Case conferences are not remunerated, and communication therefore often occurs only in written format or via third parties:

*If you want a round table, it's really very hard to organise. Physicians are self-employed, we are self-employed, [and] everyone wants this time to be paid, of course. [...] The only contact is sometimes a short phone call or [...] via our written reports [...] and that is often not enough. (Speech and language therapist, ID85, §39)*

#### *8. Partnerships and resources*

The HCPs described successful collaboration as requiring mutual respect for the expertise of the different professionals and a commitment to developing common treatment plans. Moreover, the HCPs stated that learning from other stakeholders is important to achieve treatment goals and ensure improvements. A quote from a head nurse regarding therapeutic exercises illustrates this assertion:

*[It is not enough] if the physiotherapist comes three times a week for 20 minutes. These are also things that the nursing staff can – and perhaps must be able to – take over. [...] All other professionals provide treatment on a temporary basis, and the nurse is on site 24 hours a day. (Head nurse, ID7, §73-75)*

Another key issue concerns financing. The HCPs described the fragile situation between reducing costs and delivering high-quality care for people receiving HMV. In this regard, the HCPs criticised the point that cost reduction strategies (e.g., flat-rate payments for respiratory equipment or insufficient remuneration of medical home visits) can decrease quality of care and even increase long-term costs (e.g., due to preventable infections, unnecessary transport or hospital stays).

### III. Vision and leadership

#### *9. Quality improvement-focused leadership*

The HCPs emphasised that successful leadership includes a clear vision and an understandable strategy in delivering person-centred care for people on HMV. This vision should be clearly communicated to all involved stakeholders:

*[My] focus is on ensuring that patients receive the best possible care according to their wishes and needs. I put a lot of emphasis on staff maintaining resources as well. (Head nurse, ID9, §23)*

The HCPs stated the necessity of motivating and empowering staff to share a common vision of delivering person-centred care. Therefore, the HCPs recommended leadership that supports staff in learning and developing competencies needed for highly specialised care in HMV. Moreover, the HCPs defined successful leadership as being well-known, visible, open to new ideas, and encouraging. The following quote from a nursing manager illustrates how staff can be invited to be an active part of improvement:

*We have [...] very flat hierarchies. [...] It is also important to me that I have a good relationship [...] with the employees. I want to work transparently with them [...]. I want to involve them [...] in many things, because I believe there is nothing worse than people who actually have nothing to do with the matter making the decisions. (Nursing manager, ID36, §29)*

### Capacities for improvement and relationships among the domains

The capacities for improvement are summarised in Table 3.

**Table 3:** Capacities for improvement in the quality of care for people on HMV

Key Areas	Recommendations
**I. Outcomes and impact**	• Eliminate financial disincentives to support transition management, the exploitation of rehabilitation and weaning potential, and the independent choice of long-term care setting. • Decelerate complex processes (e.g., support guideline-based transition management). • Support home visits and avoid hazardous transport and hospitalisation whenever possible. • Promote person-centred attitude and focus on the needs of patients on HMV and their families. • Provide clear information and involve all stakeholders to support shared-decision making. • Ensure staff wellbeing and support staff via supervision. • Support integrated care structures to enable interprofessional networking and collaboration. • Engage the local community, build networking structures, and implement independent counselling. • Establish mandatory quality criteria and support guideline implementation.
**II. Service delivery**	• Enable ventilated patients to be an active part of the care process. • Accompany ventilated patients on the journey through different settings (e.g., case management). • Be involved in interprofessional networks. • Implement quality insurance systems and review processes. • Ensure a supporting infrastructure to implement recognised standards and agreed-upon best practices. • Implement safety policies and support proactive risk management. • Cooperate with highly qualified nursing experts to close gaps in the provision of medical care (e.g., delegation of medical services). • Ensure that planning is flexible enough to respond to individual needs (e.g., anticipated needs for respiratory equipment). • Qualify staff to deliver safe care and ensure effective introduction, training and supervision to support staff. • Support training in family-centred care and ensure empathy and respect for the individual. • Ensure skill mixes to allow staff to learn from each other and improve internal processes. • Support interprofessional teamwork and communication (e.g., case conferences). • Ensure collaboration and develop common treatment plans to improve collaboration and outcomes.
**III. Vision and leadership**	• Share a clear vision and understandable strategy to support person-centred care. • Motivate and empower staff to be an active part of the intensive care service. • Support staff to develop competencies needed for expertise in HMV. • Encourage staff to be an active part of improvement through a visible, participatory, and open leadership.

*Abbreviations: HMV* Home mechanical ventilation

**Fig. 2:**
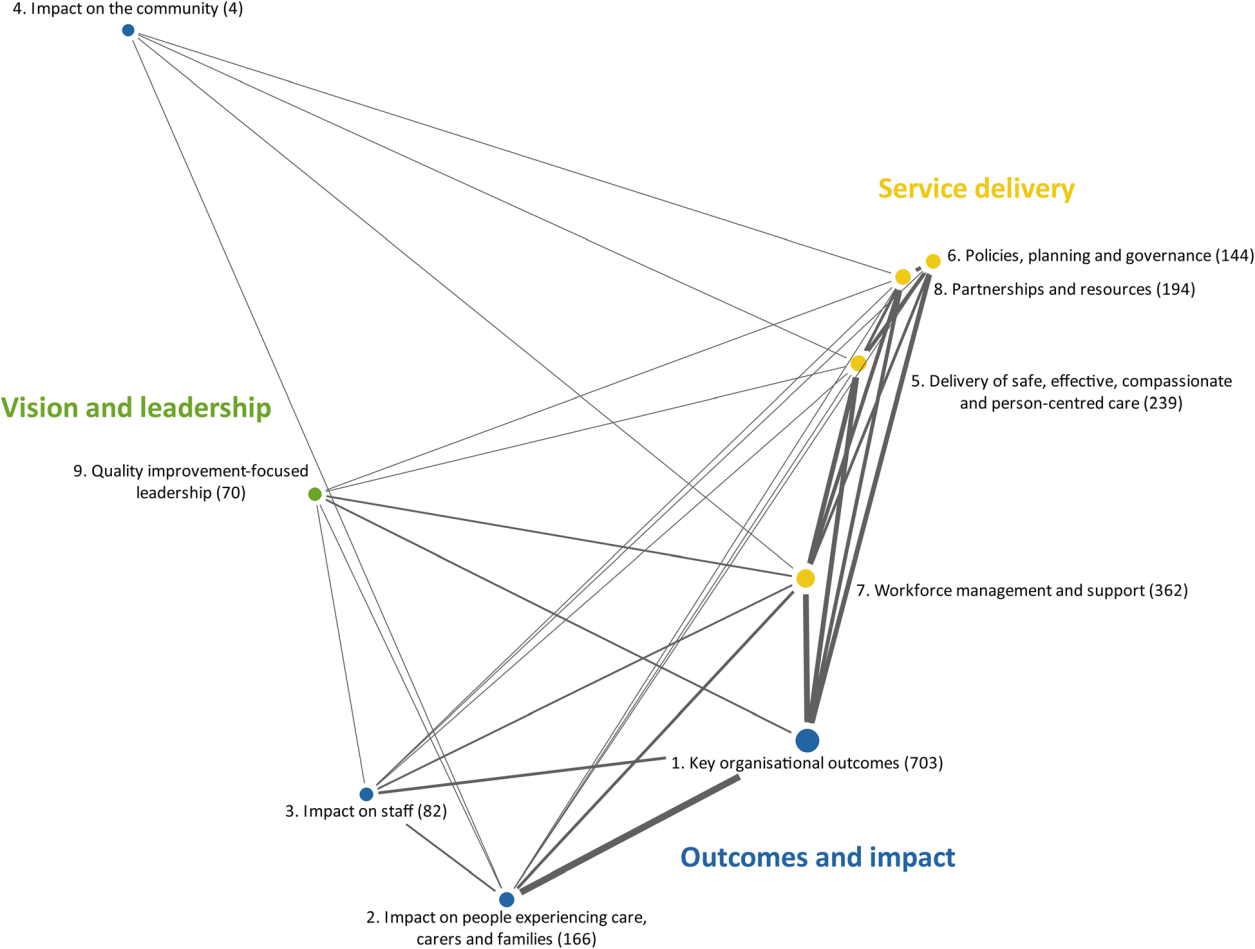
Relationships among the nine domains of the HIS quality framework. The distances between two domains represent the degree of similarity of the application of the domains in the data material (i.e., the closer the proximity, the more similar the domains are). The size of the circles symbolises the frequency of codes assigned per domain. The connecting lines between the domains indicate overlapping. The line width indicates coincidences between two domains. The numbers in brackets represent the number of meaning units per domain

Figure 2 shows the relationships among the nine domains of the quality framework. The key area *Outcomes and impact* was coded most frequently, especially the domain *(1) Key organisational outcomes* (703 meaning units). This domain had strong connections in the area of *Service delivery,* and the HCPs consistently underlined the strong impact of statutory requirements on the care of people on HMV. The domain *(4) Impact on the community* (4 meaning units) was hardly coded, since the engagement of the local community is still limited in Germany. The four domains from the key area *Service delivery* were closely connected and demonstrated the link between the processes and the management of intensive care services. The domain *(8) Workforce management and support* (362 meaning units) was coded frequently and showed numerous connections to other domains since this domain was assessed by several HCPs as highly sensitive and central to successful quality of care. The key area V*ision and leadership* was represented by the domain *(9) Quality improvement-focused leadership* (70 meaning units) and had connecting lines to many other domains. The HCPs considered a major strength for improving the quality of care in this domain.

## Discussion

Our interviews with the HCPs reveal the complex interaction between the existing health care system, the delivery of outpatient intensive care, and leadership visions influencing the quality of care for people on HMV. Overall, quality of care is described as highly heterogeneous. Our results show, that high-quality care is possible, but standardised quality criteria are necessary to ensure general compliance. The main barriers to high-quality care are driven by financial disincentives in the health care system and lead to an acceleration in transition management, a neglect of weaning potential and a shortage of skilled professionals. The central recommendations for improving the quality of service delivery are to convey a person-centred attitude through training and supervision of staff and an inspiring and participatory leadership. An integrated care structure based on medical home visits and outpatient rehabilitation should be developed to optimise HMV treatment.

The domain *(1) Key organisational outcomes* (703 meaning units) was coded most frequently in our analysis. This finding reveals that the German health care system, with its statutory requirements, has an enormous influence on the delivery of care for people on HMV. Our results confirm once again what has been criticised in outpatient intensive care in recent years [10, 13, 14]: Financial disincentives lead to mismanagement in the care of people on HMV. To counteract this problem, the German government passed the Intensive Care and Rehabilitation Strengthening Act (IPReG) in 2020 [23]. The IPReG calls for the regular assessment of weaning potential, improvement in access to rehabilitation, and mandatory quality controls. In addition, the cost-intensive and hard-to-control 1:1 care at peoples’ homes should become the exception under strict conditions, while inpatient care should become the standard by eliminating high co-payments that were previously implemented. Therefore, the IPReG has been criticised for violating the right to self-determination and autonomy of affected individuals [23]. Our interviews with the HCPs were conducted from June 2019 to May 2020, which means that more than half of the interviews were performed after the first draft of the IPReG was published in August 2019; thus, our results partly reflect the debate around the act.

The results of our study confirm the need to promote rehabilitation and weaning potential, since many patients are discharged to HMV without having been treated in a specialised weaning centre. A recent study based on the German WeanNet registry (time period: 2011 to 2015) found that 64% of 11,424 intensive care unit (ICU) patients could be successfully weaned from invasive ventilation in a specialised weaning centre (for comparison, 15% died, and 21% could not be weaned) [24]. Moreover, our study added that successful rehabilitation should be focused on the needs of ventilated patients and their families. In this regard, our results are consistent with a recent scoping review, revealing that in parallel to objective clinical data, individual patient needs and an appreciation of the physical and psychological burden of weaning are important for a successful weaning process [25]. Furthermore, it should be considered that a proportion of people on HMV would be dependent on ventilation for their entire lives due to their underlying disease (e.g., muscular dystrophies, paraplegia). These people need a long-term perspective that enables them to lead a self-determined life in their own homes [2, 8].

German guidelines for treating chronic respiratory failure [8] and prolonged weaning [26] recommend the initiation of HMV in specialised weaning centres. Although the process for a structured transition is well described [8, 26, 27], the reality in Germany deviates greatly from it: Our HCPs noted that a journey into a problematic care situation starts as early as in the hospital. This patient journey is highly influenced by accelerated processes, early discharge, unstructured transition management, and a lack of interprofessional collaboration to support shared decision making. Herein, our results are in line with a recent qualitative study with 15 experts in transition management. In this study, patients’ pathways to HMV were described as depending on chance and as being hazardous, unsafe and non-transparent [28].

Our results underline the need for outpatient rehabilitation to meet the requirements of people with long-term HMV. The HCPs recommended outpatient treatment and medical home visits to avoid hazardous transport and burdens due to the absence of trusted caregivers. Some practical projects have suggested that specialised outpatient ventilation centres for HMV would be a feasible option: A German pilot project showed that an outpatient care structure based on a tandem of a physician and a respiratory therapist could be established with benefits for patients and carers [29]. Comparable physician-respiratory therapist teams operate in German medical centres for people with disabilities (MZEB) that specialise in ventilator-dependent patients. Due to the complex treatment needs of people on HMV, this type of care could also be forward-looking [30]. Our study emphasises a further advantage of physician-nursing expert teams: The delegation of medical tasks could counteract the existing shortage of specialised physicians and strengthen the professionalisation of nursing.

Our findings highlight the association between the existing system-related shortage of professionals, its impact on staff wellbeing and the delivery of safe and person-centred care. In line with Lehmann and colleagues [10], we found that in addition to nurses, physicians and therapists specialised in HMV were urgently needed. The results of our study indicate a large difference in competence requirements for nurses caring for someone at home compared to those of nursing caring for someone in a shared living community. In particular, caring at someone’s home was experienced as challenging due to difficulties in role definition, conflicts with family members and the burden of being solely responsible for the patient. These challenges have been described in national [31] and international studies [9, 32]. Responding to the challenges of outpatient intensive care, the participating HCPs recommended a long induction period, on-the-job training, advanced education, and supervision.

What data-driven recommendations can be made for the delivery of person-centred intensive care for people on HMV? Our study found that the *delivery of safe, effective, compassionate and person-centred care* (domain 5; 239 meaning units) is possible if key processes and management are well integrated. The HCPs emphasised the importance of standardised procedures, proactive planning, effective communication and collaborative working across partnerships to ensure that processes run as intended. A recent qualitative study examined aspects of safety from the perspective of people on HMV [33] and their family caregivers [34]. Our study adds the perspective of HCPs and draws a similar conclusion: In addition to the clinical competence of nurses, the safe delivery of care requires continuity, trust and the full involvement of the people receiving care.

Since HMV should be supported by an interprofessional team of nurses, physicians, therapists, and equipment providers [8], clear responsibilities, effective communication and common treatment goals are needed. However, in our interviews, the HCPs criticised the lack of structures for interprofessional collaboration and recognised a serious need for case conferences and integrated care structures enabling collaboration. International treatment concepts could serve as models for the development and implementation of new integrated care structures [35]. German experts see the established concept of specialised outpatient palliative care (SAPV) [36] as a transferable interprofessional model for specialised outpatient intensive care for ventilated patients [37].

### Strength and limitations

Our study has clear strengths. Using the framework method [17], our study provides in-depth, holistic insights into a complex system influencing the quality of care for ventilated patients in outpatient intensive care. Although the HIS quality framework [19] is regularly used as a practice tool for self-evaluation and quality assurance, the domains and quality indicators of the framework proved to be suitable as a pre-existing theoretical structure for our deductive content analysis in the context of outpatient HMV in Germany. The framework method provided further advantages in this study, such as the ability to perform structured comparison of different care settings and identify capacities for improvement. Moreover, with the core element of person-centred care, the framework made it possible to embed the concept at all levels of the health care system, from government policy to the delivery of care for people on HMV. In addition to the strong theoretical basis, our results are based on the high level of expertise of an interprofessional HMV team, including specialised physicians, nursing managers, head nurses, registered nurses, nursing experts (i.e., instructors, consultants, and auditors), equipment providers, physiotherapists, occupational therapists, and speech and language therapists. Furthermore, many of the participating HCPs had experienced first-hand how the quality of care in HMV has changed over time (professional experience: mean 9.4 years). Our sample size was limited based on meaning saturation, which is needed to develop an understanding of the big picture and is usually reached after 16 to 24 interviews [22]. We only reached meaning saturation after 87 interviews, which reflects the complexity of the themes that emerged and the interaction of the three key areas influencing the quality of care for people on HMV in Germany.

The following limitations have to be discussed: One could argue that the quality framework, which was developed in the context of the National Health Service (NHS) of the United Kingdom (UK), is not completely transferable to the German health care system [19]. To counter to this argument, we carefully reviewed the framework before we decided to use it in our analysis. The practical application in this study proved its adaptability for the German health care system. Moreover, we recruited participants from an existing expert network and from intensive care services already involved in the OVER-BEAS project. Consequently, we must consider the risk of selection bias. It is likely that our sample consisted of HCPs with high demands on quality of care and a general interest in improvement. Thus, care deficits may have been overestimated in our results. On the other hand, it is also possible that such deficits were underestimated since we used intensive care services in participant recruitment and these services presumably asked more satisfied employees to participate in this study. Due to pragmatic reasons (e.g., long distances, no public transport in rural areas, and HCPs’ time preferences), the interviews were conducted by telephone. It is sometimes argued that the quality of telephone interviews is lower than the quality of face-to-face interviews [38], but this cannot be confirmed based on the content or scope of our interviews (mean duration of 31 min). Finally, there is the possibility of social desirability bias. Therefore, we adopted strategies such as comprehensive clarification and privacy assurance, which are recommended to minimise this problem [39]. The HCPs seemed to be honest and transparent in their criticisms; hence, we regard the risk for social desirability bias to be rather low. Nevertheless, it would be conceivable that the HCPs discussed their own activities in a more positive light and tended to criticise others. Since our results provide a broad range of experience from the perspectives of leaders, practitioners, and internal and external partners, such influences should be balanced.

## Conclusions

This study provides in-depth insights into the complex care situation of people on outpatient HMV in Germany, Bavaria from the perspective of HCPs. The quality of care in HMV is strongly influenced by the interaction of the existing health care system, the delivery of intensive care, and leadership principles. Even though HCPs describe a very heterogeneous and partly deficient care situation for people on HMV, this study presents various examples of good practice, demonstrates that high-quality care is possible and describes how it can be implemented. Our results confirm that person-centred care is a central indicator of high-quality care manifested in all three key areas of the HIS quality framework. We found that successful person-centred care initially requires the appropriate attitude to meet people’s needs (*Outcomes and impact*), the involvement of ventilated patients in all decisions related to their care (*Service delivery*), and a common vision of person-centred care provided by inspiring leadership (*Vision and leadership*). Standardised quality criteria and corresponding control mechanisms must be implemented to ensure high-quality care for all people on HMV. The results of this study could inform the development of a person-centred integrated care structure to improve the quality of care for people on HMV.

## Supplementary Information

**Additional file 1.**

## Data Availability

The datasets generated and analysed during the current study (interview transcripts and MAXQDA data) are not publicly available due to participant confidentiality considerations. Aggregate data are available from the corresponding author upon reasonable request.

## Abbreviations

COPD: Chronic obstructive pulmonary disease
HCP: Healthcare professional
HIS: Health Improvement Scotland
HMV: Home mechanical ventilation
ICU: Intensive care unit
IPReG: Intensive Care and Rehabilitation Strengthening Act
MV: Mechanical ventilation
MZEB: Medical centre for people with disabilities
NHS UK: National Health Service of the United Kingdom
OVER-BEAS: Optimising the Care of Ventilated Patients in Outpatient Intensive Care
SAPV: Specialised outpatient palliative care
SRQR: Standards for Reporting Qualitative Research
