# Supplementary material for: How to improve the quality of care for people on home mechanical ventilation from the perspective of healthcare professionals: a qualitative study
Source: BMC Health Serv Res. 2021 Aug 5;21:774. doi: 10.1186/s12913-021-06743-3 (PMC8341833; doi:10.1186/s12913-021-06743-3)
Supplement: Supplementary file 1 — Additional file 1. [file 12913_2021_6743_MOESM1_ESM.pdf]

Additional file 1

Codebook of framework analysis

This codebook provides a more in-depth description of our analysis matrix. We used the framework method [1] to guide our deductive content analysis [2]. The domains and quality indicators of the Health Improvement Scotland (HIS) quality framework [3] were used as a theoretical structure. The HIS quality framework has been developed as a tool for self-evaluation and external quality assurance including nine domains and 25 quality indicators to improve the quality of care. In this codebook, definitions and themes of each quality indicator were cited verbatim from the HIS quality framework [3]. Example quotes from the interviews with the healthcare professionals (HCP) were given to represent coding examples.

Contents

I. Outcomes and Impact

1. Key organisational outcomes 2

2. Impact on people experiencing care, carers and families 4

3. Impact on staff 4

4. Impact on the community 5

II. Delivery of Care

5. Delivery of safe, effective, compassionate and person-centred care 6

6. Policies, planning and governance 8

7. Workforce management and support 10

8. Partnerships and resources 11

III. Vision and Leadership

9. Quality improvement-focused leadership 12

References 13

I. Outcomes and Impact

**1. Key organisational outcomes**	
**1.1. Improvements in quality, outcomes and impact**	
**Definition**	This indicator describes how well the organisation performs in continually improving the quality of care that it provides and achieving the best possible outcomes for people who use its services. It also relates to how successfully the organisation spreads and sustains quality improvement activity, and the impact of this. It also refers to prevention, improving health and reducing health inequalities as core parts of the organisation to achieve the best possible outcomes for people who use its services.
**Themes**	• Improved quality of care over time.<br>• Improved outcomes.<br>• Impact of quality improvement work.<br>• Prevention, improving health and reducing health inequalities.
**Example Quotes**	*[My impression in the last years is that] well-fed and clean is somehow sufficient. [...] It's about providing care that is not yet dangerous, [...] but it's no longer about providing rehabilitation and integration for these people. (Nursing expert, ID40, §40)*
	The biggest problem [is] physician care [...]. The patient can't come to the physician's practice, that would be much too complex, this transport. And that means you need a home visit. And you need someone who is competent and knows about ventilation, swallowing disorders and all that. (Physician, ID57, §25)
	So, let's start with the biggest problem: the changes in the hospital landscape. So, the staff shortage in the clinics, the short-term transfer of patients [...]. [The] patient has to leave as quickly as possible. [...] It's much faster, less detailed. (Equipment provider, ID22, §20-22)
	Otherwise, I believe that we need better networking, that we need structural conditions that enable us to actually have such round-table discussions. (Speech and language therapist, ID79, §88)
	In the urban area there is simply [...] more know-how. But we recently had a patient [from a rural region], for example. [...] [There] was neither a speech and language therapist nor a physician to be found, who would even drive out there and take care of the lady. (Speech and language therapist, ID82, §40)

1.2. Fulfilment of statutory duties and adherences to national guidelines	
**Definition**	This indicator describes how the organisation's fulfilment of its statutory responsibilities impacts on its performance, on outcomes for the users of the services it provides, and its staff. It focuses on evaluation of financial performance based on finance data, and how finance is used to best effect in the interests of service provision. It also relates to how the organisation takes account of statutory requirements and implements relevant legislation as well as national standards and guidance to ensure that people are safe and well cared for and that their needs are met.
**Themes**	• Financial performance.<br>• Compliance with legislation.<br>• Compliance with national standards and indicators.<br>• Responsiveness to national guidance and codes of practice.
**Example Quotes**	*Until something changed in the healthcare system, and then [the patients] were [...] kicked out of the hospital. There are flat-rate payments per case, and if a patient is considered to be untreatable, he has to leave. (Registered nurse, ID69, §42)*
	I have [a patient] who is waiting [...] for a shower stool three months after moving in. I can' t formally [...] offer this person a shower at all. This is something about quality of life, that this person is not only washed in bed. (Registered nurse, ID76, §99)
	The problem is that the care services have no interest in weaning because then the customer is gone. [...] The moment the patient is recovered, they [care services] don't earn any money, and that is a very bad structure. (Physician, ID58, §33-35)
	In general, it does not work as intended in the guideline [...] at all. We don't have a specialist [weaning] centre that accepts us for monitoring. [...] They do not have the capacity to actually screen these masses of clients [...] regularly. (Nursing manager, ID35, §79)

2. Impact on people experiencing care, carers and families	
2.1. People's experience of care and the involvement of carers and families	
**Definition**	This indicator defines the quality of the experiences of people receiving care, including how well the organisation involves them to ensure the care and support they receive is individualised to meet their assessed needs, wants and wishes. It also focuses on how confident people are about the organisation and the people who provide their care and support. This indicator is also about the quality of the experiences of carers and families in respect of their appropriate involvement in the planning and delivery of care, and consideration of their needs within the care planning process.
**Themes**	• High quality care and support that is appropriate for the individual.<br>• An individual's involvement in all decisions about their care and support.<br>• Confidence in the people and the organisation providing the care and support.<br>• Treating people who experience care with respect and dignity and upholding their human rights.<br>• Assessing health improvement and inequalities as part of the care pathway.<br>• Appropriate involvement of carers and families.<br>• Support for carers and families to be involved.
**Example Quotes**	*Outpatient intensive care has a lot to do with assistance, simply understanding that someone who is fully ventilated can go to a rock concert or a restaurant or something similar, in other words, to enable participation. That is part of this service. (Nursing manager, ID34, §47)*
	The transition to the outpatient area [...] has to be managed in a completely different way in the clinic. There is a need for a medical-ethical case conference; there is a need for a serious effort to ensure that the patient experiences rehabilitation first [...]. With real information for the relatives: What is [...] possible? How ill is [...] the patient? (Nursing expert, ID41, §83)

3. Impact on staff	
3.1. The involvement of staff in the work of the organisation	
**Definition**	This indicator is about professional involvement and commitment of staff and how the organisation encourages and supports staff to feel motivated, empowered and enabled to contribute to quality improvement and development of the organisation.
**Themes**	• Engagement and motivation of staff.<br>• Staff wellbeing.
**Example Quote**	*Most treatments fail because of the psycho-social context. [...] The real issue is definitely dealing with family and relatives, the many conflicts between nurses and relatives. [...] A big shortcoming [...] is that there are [...] no structured offers like supervision. (Nursing expert, ID44, §21)*

4. Impact on the community	
**4.1. The organisation's success in working with and engaging the local community**	
**Definition**	This indicator is about the impact that the organisation has on the wider community (including the wider health community) and how confident the public is in the services it provides. This includes engaging with the local population to raise awareness about the work of the organisation and promoting preventative measures to reduce the numbers of people who might require inpatient care. This indicator also relates to how the organisation works to identify and address health inequalities. It also features how well the organisation spreads local expertise, innovative work, and good practice to allow the wider health community to learn from this.
**Themes**	• Community engagement.<br>• Public confidence in services.<br>• Recognising inequalities.
**Example Quote**	*The relatives should have the possibility to have a service point in the community or at a local level, where they can get their information. (Occupational therapist, ID46, §87)*

II. Service Delivery

5. Delivery of safe, effective, compassionate and person-centred care	
**5.1. Save delivery of care**	
**Definition**	This indicator describes the implementation of appropriate governance systems and policies to ensure a safe environment that meets people's needs and supports them to feel safe. This includes a proactive approach to the improvement of safety, reducing harm and improving reliability of care through a culture of openness, transparency and continuous learning.
**Themes**	• Safe and supportive environment that meets individual needs.<br>• Implementation of safety policies and procedures.<br>• Safety culture.
**Example Quote**	*[During 24-hour emergency service], you experience [...] a lot. Well, [...] 70 to 80 % are operating errors, and the rest are technical problems. [...] Equipment is often [incorrectly] installed. [...] The handling is [...] sometimes catastrophic. (Equipment provider, ID26, §32)*
**5.2. Assessment and management of people experiencing care**	
**Definition**	This indicator defines the quality of assessment and management of people experiencing care, including proactive identification of issues and appropriate completion of relevant documentation and handovers. The indicator also highlights the importance of empowering people who experience care to be fully involved in all decisions relating to their care and support.
**Themes**	• Appropriate assessments.<br>• Empowering people to be fully involved in all decisions relating to their care and support.<br>• Clear and accurate documentation and handovers.
**Example Quote**	*No two days are the same, that's for sure, because it's individual care. I also attach a lot of importance to that. The client determines his daily routine. [...] Whether he wants to be washed, whether he wants to get up, whether he would rather stay in bed, whether he wants to sleep, whether he wants to watch TV in bed, whatever his wish is. (Registered nurse, ID60, §16)*
**5.3. Continuity of care**	
**Definition**	This indicator is about how people are supported as they move between care settings and providers. It focuses on the effectiveness of working with partner agencies to co-ordinate care, treatment and transitions, including consideration of the person's needs, wants and wishes.
**Themes**	• Seamless journey of care throughout different settings.<br>• Joint working with partner agencies.<br>• Appropriate transfer.
**Example Quote**	*The care services are actually on site most of the time, because they have to accompany the transport. The transport of such a patient is always difficult, because [...] the staff in the ambulance is [...] not trained on the ventilators. (Physician, ID57, §21)*

**5.4. Clinical Excellence**	
**Definition**	This indicator defines the extent to which care delivery is based on nationally agreed standards, indicators or guidance and how reliably these are implemented.
**Themes**	• Use of recognised standards or agreed best practice.<br>• Monitoring reliability.<br>• Involvement in relevant networks.
**Example Quote**	*The vast majority of [...] ventilators work very well. Excellent, they are safe [...], they are operable, they are reliable [...], they are really good. (Equipment provider, ID20, §28)*
**5.5. Data for improvement and evidence-based learning**	
**Definition**	This indicator is about the quality of data collection and analysis and how this is used to support quality assurance, organisational learning and improvement.
**Themes**	• Collection and analysis of data with feedback and recording of lessons learned.<br>• Reviewing and learning from adverse events, people's care experience data and feedback, audits and evaluations.<br>• Understanding and responding to data over time to support continuous learning to inform improvement.<br>• Data sharing across and beyond the organisation.<br>• Decision making is informed by evaluation.<br>• Quality improvement outcomes made public.<br>• Review of international practice.
**Example Quote**	*We have created various quality indicators for ourselves. [...] The quality inspection guidelines of the Health Insurance Medical Service (MDK) are very good for evaluating formal quality. In my opinion, the conceptual quality results [...] from the nursing expertise as such, and here you have to create your own quality indicators. (Nursing manager, ID31, §25)*
**5.6. Quality improvement processes, systems and programmes**	
**Definition**	This indicator is about having good systems and processes in place to support quality improvement activity, implementation and evaluation.
**Themes**	• Physical and technological capacity and capability, including use of digital information and technology to support service delivery.<br>• Knowledge management.<br>• Evaluating improvement initiatives.<br>• National improvement programmes.
**Example Quote**	*We have a quality circle [...]. The quality circle consists of five employees [...], and we are always trying […] to improve the quality of care. (Head nurse, ID19, §25)*

**6. Policies, planning and governance**	
**6.1. Policies and procedures**	
**Definition**	This indicator is about the appropriateness and efficacy of systems, policies and procedures to support staff to consistently deliver safe, effective, compassionate and person-centred care.
**Themes**	• Policies and procedures in place with routine mechanisms to review and adapt as required.<br>• Supporting infrastructure to enable staff to be proactive in line with standard operating procedures.<br>• Clear information governance systems, processes and controls in place and followed.<br>• Effective knowledge management strategy.
**Example Quote**	*[As the head of the shared living community], my responsibility includes the management of the processes […]. This starts […] with the organisation of procedures, up to the monitoring of the care practice: How is the basic care for the patient carried out, staff mentoring, staff rosters, outcome quality, how is the patient, how is the staff? (Registered nurse, ID76, §35)*
**6.2. Risk management and audit**	
**Definition**	This indicator is about proactive risk management and the effectiveness of risk management systems and processes to support the safety of staff and people experiencing care.
**Themes**	• Proactive risk management.<br>• Effective risk management systems and processes.<br>• Risk-based, informed and transparent decision making.<br>• Audit plans and processes.
**Example Quote**	*There are care services where no one operates a device unless they have been intensively instructed and have mastered the device. [...] Sometimes [the] tube is simply twisted, the socket is not in properly, [...] or material has been forgotten to be reordered. [...] [It happens] very often that the employees are not well instructed in these devices. (Equipment provider, ID23, §17)*

**6.3. Assurance framework and governance committees**	
**Definition**	This indicator is about how well the organisation's governance structures ensure the delivery of safe, effective, compassionate and person-centred care.
**Themes**	• Assurance framework and appropriate governance structure in place.<br>• Accountability for performance.<br>• Clear Board roles and responsibilities.<br>• Board members kept informed of issues and involved in walkrounds or staff and stakeholder discussions.
**Example Quotes**	*The coordinator [is actually] the physician. But experience shows [...] that the coordination [...] is taken over by the care service, because they say [...], we need this and that, and the physician writes it down, but [the physician] is responsible, even if he allows the care service to dictate it to him. (Nursing expert, ID39, §59)*
	I take over the work from the medical specialist, the respiratory physician and the intensive care physician [...], but always with their supervision. The respiratory therapist [...] needs a physician who delegates this and also needs supervision. (Nursing expert, ID41, §25)
**6.4. Planning**	
**Definition**	This indicator is about how the organisation designs its services around anticipated need and plans its service delivery and workforce in proportion to this.
**Themes**	• Design of services around anticipated need.<br>• Review of processes to ensure efficient delivery.<br>• Effective communication internally and externally to plan and co-ordinate delivery of services.
**Example Quotes**	*Because [the transition] is accelerated […], there is a lack of preparation on the receiving location. In the past, you delivered the things, instructed the care service or the relatives, and then the patient arrived. Nowadays, this often takes place at the same time. (Equipment provider, ID22, §32)*
	If you now need two tube extensions a day, the provider tells you to clean the tube extension. [...] I can't wash a disposable product with water that contains, for example, secretions and bacteria [...]. Then, the [patient] has pneumonia one day later. (Head nurse, ID4, §42-44)

7. Workforce management and support	
**7.1. Staff recruitment, training and development**	
**Definition**	This indicator is about how well the organisation recruits, inducts, trains and develops staff to ensure that the workforce is appropriately skilled to deliver safe, quality care.
**Themes**	• Effective recruitment, induction, training and development.<br>• Appropriate knowledge, skills and supervision.<br>• Clear roles, responsibilities and accountabilities.
**Example Quotes**	*We [plan] a very strong and very long induction phase [...] so that everyone involved in care feels safe, i.e., the patient, relatives, and of course, the employee himself. [...] Then, we strongly focus on training and advanced education, which means that we train employees in outpatient respiratory care externally, but also, through regular quality trainings internally. (Head nurse, ID5, §19)*
	Patient observation is the be-all and end-all. [...] I have to rely solely on my trained eye to recognize whether the patient is well or not. And I just have to [...] recognize - does she grimace, what are the facial expressions, does she have blue lips - [...] focus [...] on the basic symptoms. In the intensive care unit (ICU) [...] there is a lot of reliance on technology instead of looking at the patient. [...] In the outpatient intensive care, I am dependent on recognizing these symptoms. (Head nurse, ID10, §67)
	In the outpatient intensive care context, [...] the patient [is] not ill alone; [...] the relatives and families [must] be involved in the care [...]. And then it's a lot about the themes of proximity and distance; mindful, appreciative interaction with relatives and families; [...] feeling like a guest in the [patient's] home. (Nursing expert, ID44, §17)
**7.2. Workforce planning, monitoring and development**	
**Definition**	This indicator is about how effectively the organisation undertakes workforce planning to ensure appropriate staffing levels and skills mix that matches service requirements.
**Themes**	• Care and support is provided by the right number of people who are trained, competent and skilled to do so and follow organisational and professional codes.<br>• Regular review of resources, staff skills, skill mix and staff performance.<br>• Proactive and robust workforce planning and deployment of staff, including effective rostering and safe use of supplementary staff.<br>• Use of mandated workforce tools to ensure safe and effective staffing.<br>• Flexibility to respond to broader factors.
**Example Quote**	*[It is important], that you have a team with some people who know what they are doing. [...] We have a total of four respiratory therapists, [...] [who are] in different shared living communities. We do the nursing rounds in all the shared living communities every quarter. [...] And we simply decide what we can improve. (Registered nurse, ID76, §36)*

7.3. Communication and team working	
**Definition**	This indicator is about the effectiveness of communication between all levels of staff and with partner organisations to ensure safe, effective, compassionate and person-centred care delivery.
**Themes**	• Effective communication.<br>• Shared ownership of the challenges and solutions.<br>• Multidisciplinary team working
**Example Quotes**	*The great thing is [...] the interprofessional team; everyone contributes something new and says, "Watch out, we can take a look at that." [...] I as a physiotherapist [...], the speech and language therapist [...], as well as the nursing staff [...] who are much closer to the patient, [...] you can involve [...] them as co-therapists. (Physiotherapist, ID52, §19)*
	If you want a round table, it's really very hard to organise. Physicians are self-employed, we are self-employed, [and] everyone wants this time to be paid, of course. [...] The only contact is sometimes a short phone call or [...] via our written reports [...] and that is often not enough. (Speech and language therapist, ID85, §39)
	I'm actually a team player, [in 1:1 care] you don't really have any team contact, especially if the handover after a 12-hour-shift [is conducted] with a relative instead of the other person [of the nursing team]. (Registered nurse, ID73, §43)

8. Partnerships and resources	
**8.1. Collaborating and influencing**	
**Definition**	This indicator is about the inward and outward focus of leadership and mutual respect to enhance collaborative working across partnerships.
**Themes**	• Leadership respected by staff, stakeholders and communities.<br>• Proactive and effective working with stakeholders.<br>• Collaboration leading to improved outcomes.
**Example Quote**	*I think that the [therapists] also appreciate it, if one seeks interprofessional collaboration. And they are usually very interested in [...] joint assessment of the patient and the development of optimal care [...]. I have actually had very positive experiences with good outcomes. (Speech and language therapist, ID86, §61)*
**8.2. Cost effectiveness and efficiency**	
**Definition**	This indicator is about how the organisation makes best use of its resources.
**Themes**	• Reviews of cost effectiveness and efficiencies.<br>• Identifying and reducing unwarranted variation and waste.
**Example Quote**	*I work for a home care company, and they also want to make profits. [...] Of course, I can't [deliver] everything that someone wants or that a care service might also want. (Equipment provider, ID21, §49)*

8.3. Sharing intelligence	
**Definition**	This indicator is about mechanisms in place to ensure that learning is spread throughout the organisation and intelligence is shared with relevant external stakeholders and partner organisations in the interests of safety.
**Themes**	• Mechanisms to share relevant intelligence, learning and good practice internally and externally.
**Example Quote**	*[It is not enough] if the physiotherapist comes three times a week for 20 minutes. These are also things that the nursing staff can – and perhaps must be able to – take over. [...] All other professionals provide treatment on a temporary basis, and the nurse is on site 24 hours a day. (Head nurse, ID7, §73-75)*

III. Vision and Leadership

9. Quality improvement-focused leadership	
**9.1. Vision and strategic direction**	
**Definition**	This indicator is about the organisation having a clear vision and aims which are known and understood within and outwith the organisation.
**Themes**	• Clear strategy (values, purpose and vision) that reflects effective collaboration with stakeholders and is underpinned by quality improvement and adequate resources to support delivery.<br>• Common and clearly communicated objectives and anticipated outcomes.<br>• Board members actively influence and drive policy and strategy.<br>• Comprehensive strategic planning with Integration Joint Boards and Community Planning Partnerships that involves the public, staff and local agencies.
**Example Quote**	*[My] focus is on ensuring that patients receive the best possible care according to their wishes and needs. I put a lot of emphasis on staff maintaining resources as well. (Head nurse, ID9, §23)*
**9.2. Motivation and inspiring leadership**	
**Definition**	This indicator is about how well leadership in the organisation empowers and motivates staff.
**Themes**	• Positive and inspiring leadership.<br>• Staff encouraged to be responsibly proactive and innovative.<br>• Open and fair culture that focuses on the system and context.<br>• The organisation is well led and managed.
**Example Quote**	*I always say to my team, I like to give you the red thread, but my red thread is not set in concrete. You're welcome to go left and right, but I just want to have a structure in it [...]. And I'm actually [...] going along quite well with that. (Head nurse, ID10, §81)*

9.3. Developing people	
**Definition**	This indicator is about leaders giving staff every reasonable opportunity to develop and increase their ability to contribute to the aims of the organisation.
**Themes**	• Staff autonomy and accountability.<br>• The organisation prioritises and supports quality improvement capability building at a local and national level, and supports staff to implement improvements.<br>• Positive attitudes towards learning and improving.<br>• Staff respond effectively to complaints and adverse events, and receive feedback on them.
**Example Quote**	*Training, training again and again, refresher courses. [...] We have a very good education program, especially in intensive care, and the employees are really trained every year again and again. (Head nurse, ID9, §45)*

9.4. Leadership of improvement and change	
**Definition**	This indicator is about how leadership encourages an improvement culture and supports staff to be innovative to improve services locally.
**Themes**	• Known, visible and participatory leadership.<br>• Staff supported to identify opportunities for improvement and take ownership of improvement projects.<br>• Sufficient, knowledgeable staff to drive change and improvement.<br>• Leadership seeks out good practice and learning.<br>• Leadership is open to new ideas and responsive to challenges or obstacles and works to address sustainability issues.
**Example Quote**	*We have [...] very flat hierarchies. [...] It is also important to me that I have a good relationship [...] with the employees. I want to work transparently with them [...]. I want to involve them [...] in many things, because I believe there is nothing worse than people who actually have nothing to do with the matter making the decisions. (Nursing manager, ID36, §29)*

References

1. Gale NK, Heath G, Cameron E, Rashid S, Redwood S. Using the framework method for the analysis of qualitative data in multi-disciplinary health research. BMC Med Res Methodol. 2013;13:117. doi:10.1186/1471-2288-13-117.
2. Kyngäs H, Kaakinen P. Deductive Content Analysis. In: Kyngäs H, Mikkonen K, Kääriäinen M, editors. The Application of Content Analysis in Nursing Science Research. Cham: Springer International Publishing; 2020. p. 23–30. doi:10.1007/978-3-030-30199-6_3.
3. Health Improvement Scotland. Quality of Care Approach. 2018. http://www.healthcareimprovementscotland.org/our_work/governance_and_assurance/quality_of_care_approach/quality_framework.aspx. Accessed 6 Nov 2020.
